# A novel replication-deficient FCV vaccine provides strong immune protection in cats

**DOI:** 10.1128/jvi.00093-25

**Published:** 2025-07-08

**Authors:** Wuchang Heng, Dan Zang, Ruiyu Li, Qian Jiang, Jiasen Liu, Honglin Jia, Hongtao Kang

**Affiliations:** 1 State Key Laboratory for Animal Disease Control and Prevention, Harbin Veterinary Research Institute, Chinese Academy of Agricultural Sciences 111613 , Harbin, China; 2 Heilongjiang Research Center for Veterinary Biopharmaceutical Technology, Harbin Veterinary Research Institute, Chinese Academy of Agricultural Sciences 111613 , Harbin, China; University of Michigan Medical School, Ann Arbor, Michigan, USA

**Keywords:** replication-deficient FCV, vaccine, calicivirus

## Abstract

Feline calicivirus (FCV) belongs to the family Caliciviridae, which includes important animal and human pathogens such as human norovirus and rabbit hemorrhagic disease virus. The fast evolution and great diversity of FCV make it highly difficult to develop vaccines that can induce sterilizing immunity. In this study, we evaluated the efficacy of a vaccine candidate generated via a replication-deficient vaccine strategy. Immunization with the vaccine generated a high level of neutralizing antibodies and highly reduced clinical outcomes against the challenge with a homologous virulent systemic feline calicivirus (VS-FCV) strain. Moreover, the VP1 gene of the vaccine was replaced with that derived from a heterologous virus strain. Vaccination with two combined virus constructs containing genetically distant VP1 genes led to the generation of broad neutralizing antibodies against FCV strains in cats. Notably, this replication-deficient FCV has the potential to serve as a viral vector vaccine, enabling the delivery of a foreign gene with stable genetics. In summary, this vaccine strategy holds great promise for developing safe, effective, and multivalent vaccines to prevent and control calicivirus infection.

**IMPORTANCE:**

FCV is one of the leading causes of respiratory diseases in cats. Over the last 20 years, certain strains evolved into VS-FCV, with severe symptoms and increased fatality. Updating and developing vaccines promptly are essential. Here, we employed reverse genetics to partially delete the VP2 gene of FCV, rescuing the replication-deficient vaccine candidate rHBDL2 FCV-△VP2. Immunization with this candidate generated high levels of neutralizing antibodies against FCV strains and significantly reduced clinical symptoms. Furthermore, the adaptability of this replication-defective FCV platform holds potential for the development of viral vector vaccines as well as multivalent vaccines, which are also crucial for the prevention and control of other calicivirus infections.

## INTRODUCTION

Caliciviridae is a family of single-stranded, positive-sense RNA viruses that are 27–40 nm in diameter (1) and harbor a 7.4–8.3 kb genome in icosahedral capsids (2). Caliciviridae comprises five well-known genera (Vesivirus, Lagovirus, Norovirus, Sapovirus, and Nebovirus) and six newly discovered genera (Bavovirus, Minovirus, Nacovirus, Recovirus, Salovirus, and Valovirus) (3). These viruses, which have a broad host range, can infect mammals, fish, and birds, posing significant threats to both human and animal health. The cultivation of most caliciviruses *in vitro* presents significant challenges. Currently, there is no highly efficient and user-friendly cell culture model available for human norovirus (HuNoV), which causes over 20% of acute gastroenteritis cases (4); however, recent research has shown that HuNoV replication is limited to stem cell-derived enteroids (5). In addition, the lack of ideal animal models further hinders research on Caliciviridae biology and vaccine development (6). Feline calicivirus (FCV) and murine norovirus (MNV) are among the few viruses in Caliciviridae that can be cultured *in vitro*. In particular, FCV is highly pathogenic in animals and has been used as a model for studying the biology and pathogenic mechanisms of other caliciviruses (2, 7).

FCV is prevalent among cat populations (8) and has a range of clinical manifestations. These manifestations include upper respiratory tract symptoms, tongue ulcers, gingivostomatitis, and lameness syndrome. Over the last 20 years, certain strains have evolved into virulent systemic feline caliciviruses (VS-FCVs) with highly effective replication properties which can cause alopecia; skin ulcers; oral ulcers; auricular, nostril, and necrotizing pododermatitis with serum cell scabs; and other symptoms, including subcutaneous edema; bronchointerstitial pneumonia; and pancreatic, hepatic, and splenic necrosis, resulting in a relatively high mortality rate in infected cats (9–15). The primary factor underlying this situation is the RNA polymerase of FCV, which lacks sequence proofreading capabilities and exhibits low fidelity. This feature results in a high mutation rate and the emergence of multiple genotypes under immune pressure. Consequently, FCV strains can easily evade immune clearance, and existing vaccines are not completely effective (16–19). As one of the primary companion animals, cats are closely associated with humans, and there are more than 600 million cats worldwide (20). The development of novel FCV vaccines is crucial for the prevention and control of FCV infection.

The FCV genome is approximately 7.7 kb in length and encodes six non-structural proteins (VPg, P5.6, PP, P30, P32, and P39) and two structural proteins (VP1 and VP2) (21), with the virion capsid consisting of 180 copies of the major structural protein VP1 and 12 copies of the low-copy minor structural protein VP2 (22). FCV interacts with the feline junctional adhesion molecule 1 (fJAM-A) functional receptor and then enters target cells via clathrin-mediated endocytosis before releasing genomic RNA (gRNA) into the cytoplasm (23–25). A previous study showed that VP2 N-terminal deletion prevented the production of infectious virions and that infectivity was restored upon transient expression of the back-complemented full-length VP2 protein *in vitro* (26). The cryo-EM structure of FCV (F9 strain) in complex with fJAM-A suggests that the receptor interacts with the VP1 protein, which triggers the formation of a large portal-like structure made up of several VP2 proteins that may serve as a gateway for the calicivirus genome to enter the host cell cytoplasm through the endosomal membrane, thereby triggering infection (2). Confirming this hypothesis in recent studies, FCVs use this VP2 protein structure to penetrate the endosomal membrane to release the genome, and hydrophobicity at the N-terminus of the VP2 protein plays a crucial role in the infection process (22).

The development of new vaccines is essential because of the rapid evolution and great diversity of viruses. Previous studies have shown that constructing replication-defective Caliciviridae family viruses is feasible (26, 27). In this study, we evaluated the protective efficacy of a replication-defective FCV vaccine candidate (FCV-△VP2), which was rescued by utilizing a complementing cell line stably expressing the VP2 protein, and assessed its potential as a vaccine vector for delivering foreign antigens. Our data indicated that recombinant FCV-△VP2 is a promising vaccine candidate and is capable of serving as a vaccine vector.

## RESULTS

### Rescue and characterization of rHBDL2 FCV-△VP2

**Fig 1:**
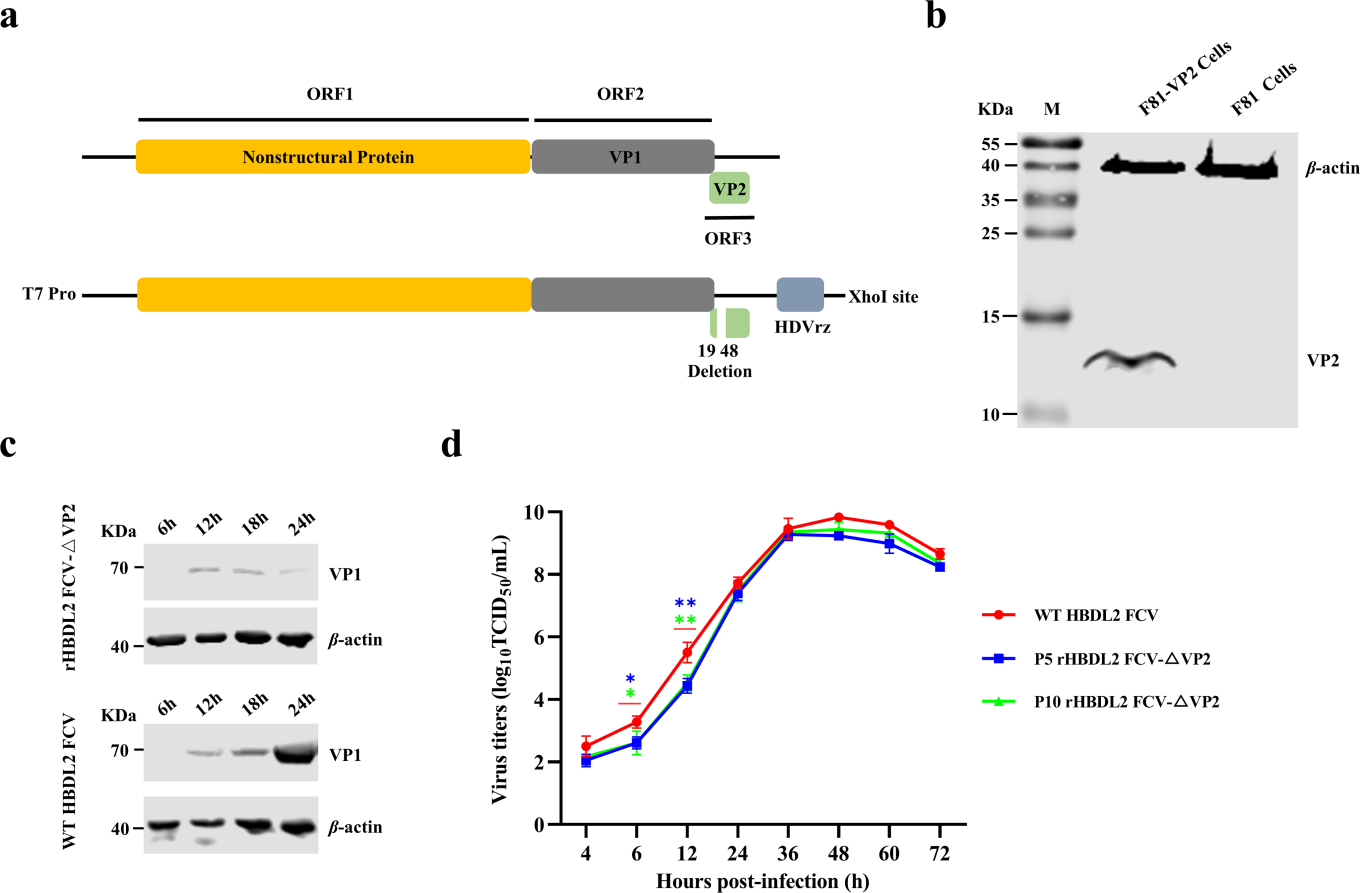
Rescue and characterization of rHBDL2 FCV-△VP2. (**a**) Schematic representation illustrating the construction of infectious clones of rHBDL2 FCV-△VP2, featuring a targeted deletion of nucleotides 19–48 at the N-terminus of the FCV VP2 gene. (**b**) rHBDL2 FCV-△VP2 was successfully rescued in a cell line stably expressing the VP2 protein (F81-VP2 cells). (**c**) Conventional F81 cells were infected with either rHBDL2 FCV-△VP2 or wild-type HBDL2 FCV at an MOI of 0.001, and Western blot analysis was conducted to assess the VP1 protein expression levels at 6, 12, 18, and 24 h post-infection, with β-actin used as an internal control. (**d**) The viral titers (TCID_50_) were assessed at 4, 6, 12, 24, 36, 48, 60, and 72 h post-infection to construct a growth curve for p5 and p10 rHBDL2 FCV-△VP2, as well as WT HBDL2 FCV (MOI = 0.001), in F81-VP2 cells. Statistical analysis was conducted via one-way ANOVA with Tukey’s multiple comparison test, with significance levels denoted as follows: *P <  0.05, **P < 0.01. The color-coded asterisk (*) and underline (_) denote comparisons between viral strains.

The rHBDL2 FCV-△VP2 infectious clone cDNA was constructed by inserting a T7 promoter at the 5′ end and an HdvRz ribozyme sequence at the 3′ end of the FCV genome sequence, accompanied by deletion of the viral VP2 N-terminus at the 19–48th nt (Fig. 1a). The synthesized capped RNA was subsequently transfected *in vitro* into the F81-VP2 cell line, which stably expresses VP2 (Fig. 1b), to rescue the recombinant virus rHBDL2 FCV-△VP2. The replication capacity of rHBDL2 FCV-△VP2 in normal F81 cells was assessed by measuring VP1 protein expression. Compared with that observed post-infection with wild-type (WT) HBDL2 FCV, a minimal level of VP1 expression was observed at 12 h post-infection with rHBDL2 FCV-△VP2. Furthermore, VP1 expression did not increase at the 12 h mark but subsequently decreased at 24 h (Fig. 1c). These findings suggest that rHBDL2 FCV-△VP2 is unable to replicate in normal F81 cells.

To examine the replication capacity of rHBDL2 FCV-△VP2 in F81-VP2 cells, the virus was propagated to the P10 generation (no mutations following the whole genome sequencing) at a multiplicity of infection (MOI) of 0.01. Growth curves for F81-VP2 cells infected with rHBDL2 FCV-△VP2 and WT HBDL2 FCV at an MOI of 0.001 were assessed at the P5 and P10 generations. Measurements were taken specifically at 4, 6, 12, 24, 36, 48, 60, and 72 h post-infection. Quantitative analysis revealed comparable replication kinetics between the recombinant and wild-type viruses, with statistically significant differences observed only at 6 and 12 h post-infection. The recombinant virus achieved peak titers of 10^9.43^ 50% tissue culture infectious dose (TCID_50_)/mL, demonstrating replication efficiency close to the WT strain (Fig. 1d). These data demonstrated that the deletion of nucleotides 19–48 at the N-terminus of VP2 in the FCV gRNA resulted in the virus losing its ability to replicate in conventional cell lines, which is consistent with a previous report (26). Notably, we successfully obtained a replication-deficient recombinant virus with high viral titers that was capable of replicating exclusively in the F81-VP2 cell line.

### N-terminal deletion of the FCV VP2 protein inhibited viral genome replication but did not impede virion assembly

**Fig 2:**
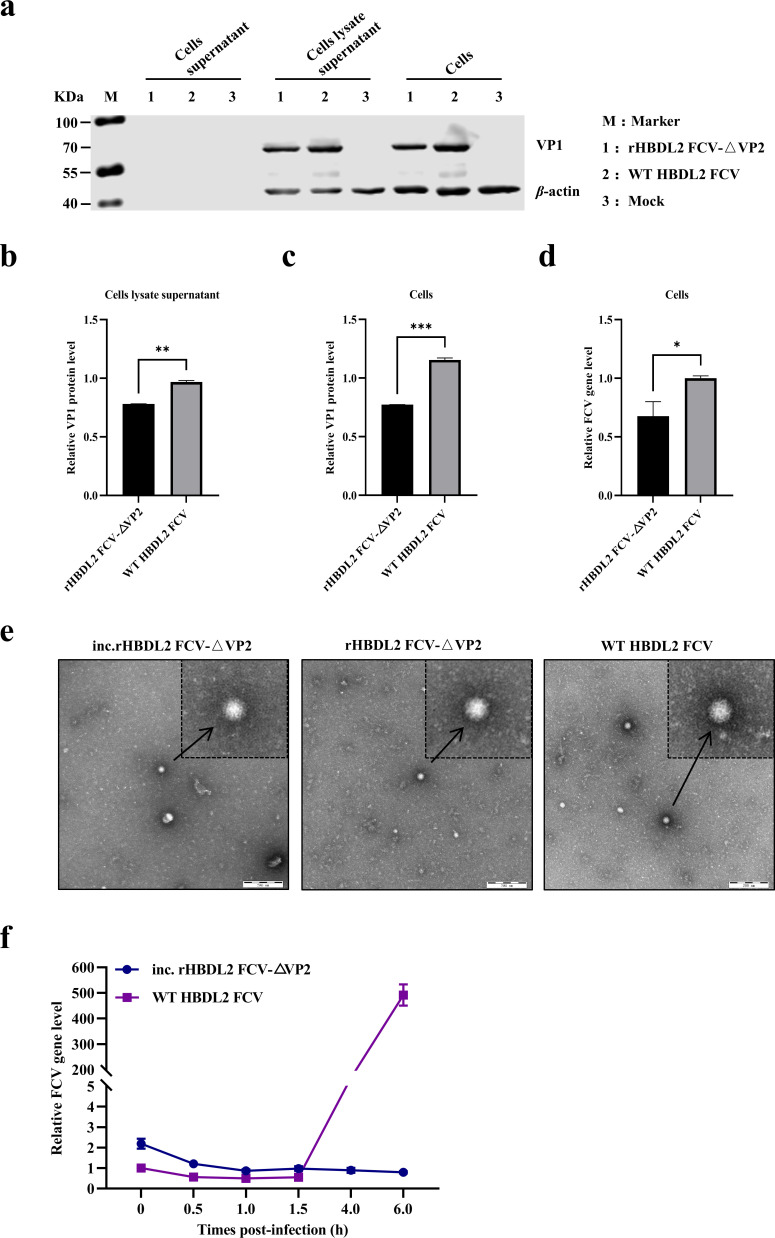
N-terminal deletion of the FCV VP2 protein inhibited viral genome replication without impeding virion assembly. (**a**) Conventional F81 cells were infected with either rHBDL2 FCV-△VP2 or WT HBDL2 FCV at an MOI of 50. Western blot analysis was conducted to assess the levels of the VP1 protein in the cell supernatant, the cell lysate supernatant, and within the cells at 6 h post-infection, with β-actin used as an internal control. Quantitative analysis of the gray values was performed to measure the bands corresponding to the VP1 protein in the cell lysate supernatant (**b**) and within the cells (**c**). Additionally, RT-qPCR analysis was employed to determine the relative levels of the FCV genome within the cells (**d**). (**e**) Transmission electron microscopy was used to observe the morphology of the inc.rHBDL2 FCV-△VP2, rHBDL2 FCV-△VP2, and WT HBDL2 FCV viral particles, as indicated by black arrows. (**f**) The relative genomic levels of FCV in conventional F81 cells infected with inc.rHBDL2 FCV-△VP2 and WT HBDL2 FCV were quantified via RT‒qPCR at 0, 0.5, 1.0, 1.5, 4.0, and 6.0 h. Statistical analysis was conducted via unpaired *t*-tests, with significance denoted as *P < 0.05, **P < 0.01, and ***P < 0.001.

To elucidate the mechanism underlying the replication defects of rHBDL2 FCV-△VP2 in normal F81 cells, rHBDL2 FCV-△VP2 and WT HBDL2 FCV were used to infect the cells at a high MOI of 50. Following a 6 h incubation period, cytopathic effects (CPEs) were evaluated comprehensively. The level of the VP1 protein in the cell supernatant, cell lysate supernatant, and precipitated cells was analyzed via Western blotting. The VP1 protein was not detected in the cell supernatant. However, the VP1 protein was detected in both the cell lysate and the precipitate. The level of the rHBDL2 FCV-△VP2 VP1 protein was lower than that of the WT HBDL2 FCV (Fig. 2a through c), and reverse transcription quantitative PCR (RT-qPCR) analysis of the FCV genome further revealed that, compared with that of WT HBDL2, the copy number of rHBDL2 FCV-△VP2 was reduced (Fig. 2d). These findings suggest that both rHBDL2 FCV and WT HBDL2 completed only single-cycle replication within cells. Compared with the WT HBDL2 FCV, the rHBDL2 FCV containing intact and partial VP2 exhibited diminished replication efficiency. The viral particles of incomplete rHBDL2 FCV-△VP2 (inc.rHBDL2 FCV-△VP2), which were produced by rHBDL2 FCV-△VP2 in normal F81 cells, were subsequently analyzed via transmission electron microscopy. The results indicated that the morphological characteristics of the viral particles closely resembled WT HBDL2 FCV particles (Fig. 2e), suggesting that the N-terminal deletion of the VP2 protein does not impact virion assembly. Finally, semiquantitative analysis was employed to evaluate the genome levels of FCVs between the inc.rHBDL2 FCV-△VP2 and WT HBDL2 FCVs in normal F81 cells. These two viruses were inoculated into cells, adsorbed at 4°C for 1 h, and then incubated at 37°C. Total cellular RNA was extracted at different times to assess the FCV genome. The results indicated that the genomic levels of inc.rHBDL2 FCV-△VP2 tended to decrease during the adsorption and internalization phase (0–1.5 h), which was similar to the trend in the WT HBDL2 FCV. After 1.5 h, the genome level of WT HBDL2 FCV increased rapidly, whereas that of inc.rHBDL2 FCV-△VP2 decreased significantly (Fig. 2f). This finding suggests that the N-terminal deletion of the VP2 protein in inc.rHBDL2 FCV-△VP2 virions impairs their replication capability, allowing only a single infection cycle in regular F81 cells, with stable replication occurring only in F81-VP2 cells.

### rHBDL2 FCV-△VP2 produces high levels of neutralizing antibodies

**Fig 3:**
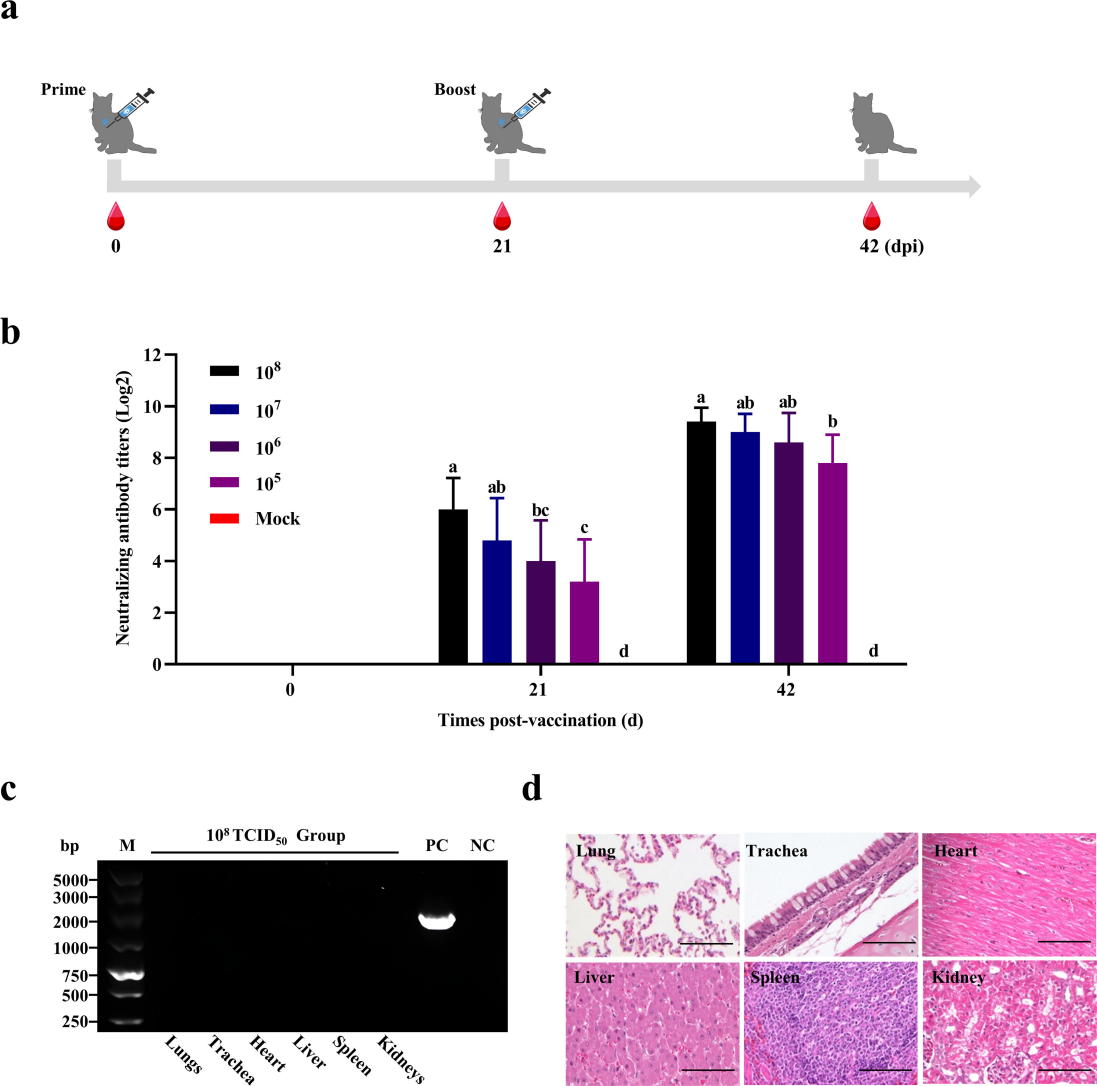
rHBDL2 FCV-△VP2 produced high levels of neutralizing antibodies. (**a**) rHBDL2 FCV-△VP2 was administered via intramuscular injection to immunize cats at different viral titers: 10^8^ TCID_50_/mL (*n* = 6), 10^7^ TCID_50_/mL (*n* = 5), 10^6^ TCID_50_/mL (*n* = 5), 10^5^ TCID_50_/mL (*n* = 5), and DMEM control (*n* = 5). The immunization was performed twice, with an immunization cycle of 42 days. (**b**) The levels of neutralizing antibodies were assessed after the primary and booster immunizations. Groups with at least one same letter indicate no statistical differences, while groups marked with different letters indicate significant differences (*P* < 0.05). (**c**) After the completion of the immunization cycle, one cat from the 10^8^ TCID_50_/mL immunization group was randomly selected, and heart, liver, spleen, lung, and kidney samples were collected for PCR testing using specific primers targeting rHBDL2 FCV-△VP2, with the viral genome serving as a positive control and sterile water as a negative control. (**d**) Histopathological examination of HE-stained sections of major organ tissues post-immunization of 10^8^ TCID_50_/mL rHBDL2 FCV-△VP2. Scale bar: 100 µm.

To evaluate the efficacy of rHBDL2 FCV-△VP2 in eliciting anti-FCV antibody production, cats were subjected to two immunizations with varying doses of rHBDL2 FCV-△VP2. Blood samples were collected 21 days after each immunization to assess neutralizing antibody titers (Fig. 3a). Seroconversion was observed in all cats by day 21 after initial immunization in the 10^8^ and 10^7^ TCID_50_/mL dose groups. In contrast, seroconversion rates were 80% in the 10^6^ TCID_50_/mL group and 60% in the 10^5^ TCID_50_/mL group following the initial immunization. Notably, the mean neutralizing antibody titer in the 10^8^ TCID_50_/mL immunization group reached 1:2^6^ prior to booster immunization. The booster immunization resulted in an increase in neutralizing antibody titers across all immunization groups within 3 weeks after immunization. The mean neutralizing antibody titers ranged from 1:2^7.8^ to 1:2^9.4^ among the groups (Fig. 3b). No adverse reactions were observed in any of the cats within the immunization group following immunization. After the immunization, one cat was randomly selected from the group immunized with 10^8^ TCID_50_/mL for further analysis. This cat was sacrificed, and its trachea, heart, lung, and kidney tissues were homogenized for PCR detection. The PCR results indicated that all the tissues tested negative (Fig. 3c). Histopathological examination revealed no alterations in the primary organs, including the lungs, trachea, heart, liver, spleen, and kidneys (Fig. 3d). These findings demonstrate that rHBDL2 FCV-△VP2 immunization effectively induces anti-FCV neutralizing antibody responses in cats and has a favorable safety profile.

### rHBDL2 FCV-△VP2 provides robust immune protection in cats

**Fig 4:**
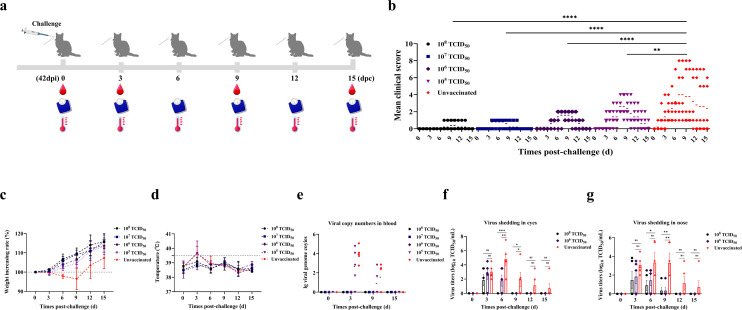
rHBDL2 FCV-△VP2 provides robust immune protection in cats. (**a**) All cats were challenged with 5 × 10^7^ TCID_50_ WT HBDL2 FCV. The experiment included five cats per group, and the challenge period was extended to 15 days. Clinical symptom scores (**b**) were compiled, and changes in weight (**c**) and temperature (**d**) were systematically recorded. (**e**) Anticoagulated blood samples were collected at 0, 3, 9, and 15 days post-challenge (DPC; *n* = 5 at each time point) for the quantification of viral RNA copies via RT-qPCR. Ocular (**f**) and nasal (**g**) swab samples were collected at 0, 3, 6, 9, 12, and 15 DPC (*n* = 5 per time point) to determine viral titers (TCID_50_). Statistical analyses were conducted via unpaired *t*-tests (**b**) and one-way ANOVA with Tukey’s multiple comparison test (**f and g**), with significance levels denoted as follows: *P <  0.05, **P  < 0.01, ***P < 0.001, ****P < 0.0001. ns, non-significant.

**Fig 5:**
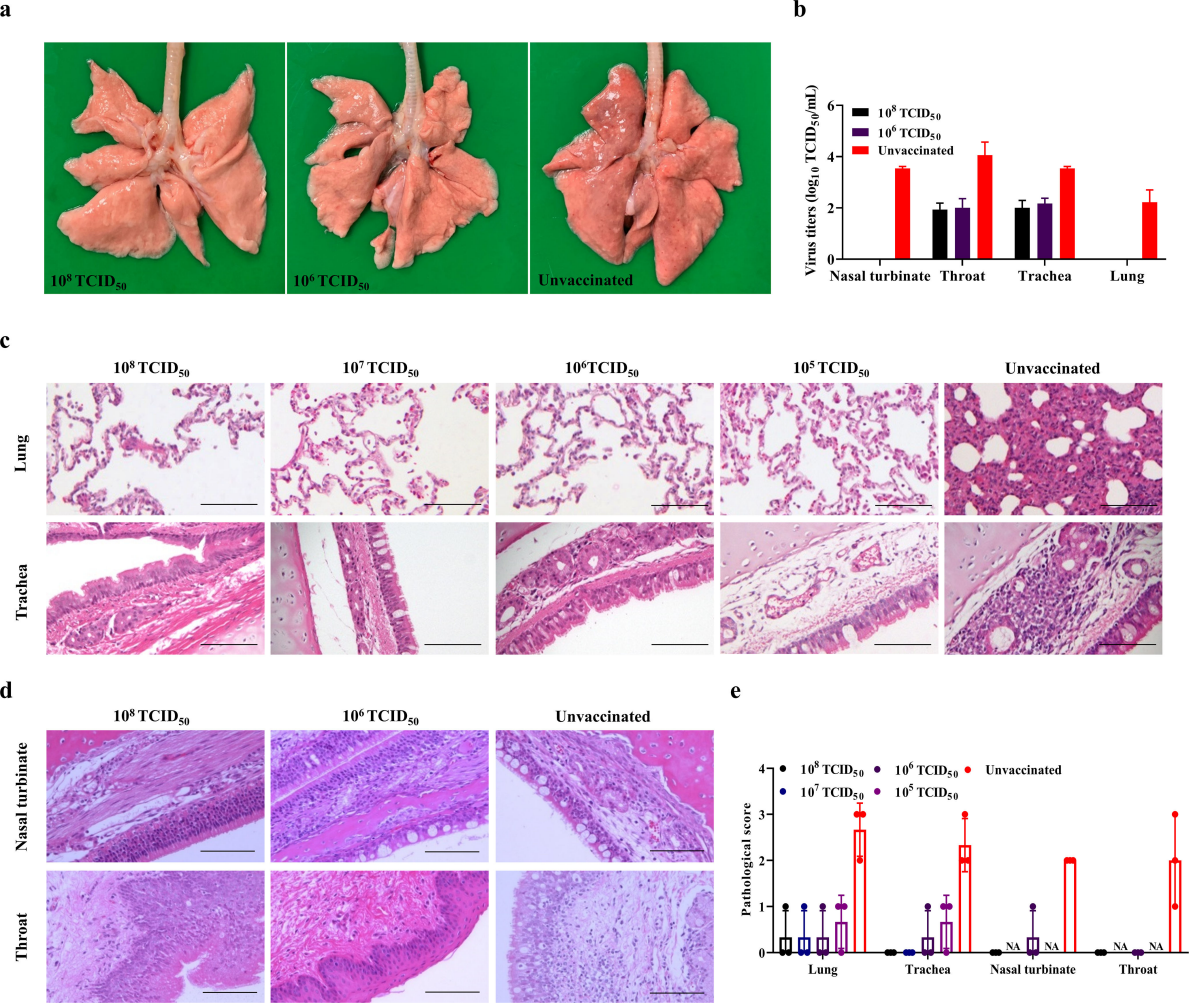
rHBDL2 FCV-△VP2 reduces FCV invasion and pathological damage to tissues. (**a**) Cats were euthanized at 15 DPC, and lungs were photographed for gross observation after necropsy. (**b**) At 15 DPC, cats from the immunization group and the unvaccinated control group were randomly selected to measure viral titers (TCID_50_) in nasal turbinates, throat, trachea, and lung tissues. (**c**) Histopathological examination of HE-stained lung and trachea tissue sections following VS-FCV exposure. Scale bar: 100 µm. (**d**) Histopathological examination of HE-stained nasal turbinate and throat tissue sections following VS-FCV exposure. Scale bar: 100 µm. (**e**) Histopathological scoring for the lung, trachea, nasal turbinate, and throat of cats: 0, normal; 1, mild; 2, moderate; 3, severe. NA, not available.

The ability of the rHBDL2 FCV-△VP2 to elicit a protective immune response against VS-FCV challenge was assessed in cats. All the cats, divided into five groups, were challenged with the homologous FCV HBDL2 strain via intranasal and ocular administration 3 weeks after booster immunization. Clinical symptoms were recorded daily over a 15 day period, while body temperature and body weight were monitored every 3 days. Venous blood samples were collected on days 0, 3, 9, and 15 for RNA extraction for RT-qPCR analysis (Fig. 4a). In the unvaccinated control group, 80% of the cats presented typical clinical signs of FCV infection, and the clinical scores for this group were significantly higher than those reported for the rHBDL2 FCV-△VP2-immunized groups (Fig. 4b). Most cats in the immunized groups presented no clinical signs of infection; only a few cats in the 10^5^ TCID_50_/mL immunization group presented mild clinical symptoms. The body weight of the vaccinated group consistently increased, whereas that of the unvaccinated control group tended to decrease from days 0 to 9, followed by a recovery in weight from days 9 to 15 (Fig. 4c). In terms of body temperature, the groups vaccinated with 10^8^, 10^7^, or 10^6^ TCID_50_/mL presented fluctuations within the normal range. Conversely, the groups vaccinated with 10^5^ TCID_50_/mL, and the unvaccinated control group experienced a temporary increase in body temperature exceeding 39.5°C during the initial 3 days post-challenge, with a subsequent return to normal levels after this period (Fig. 4d). In the blood samples, no viral copy numbers were detected in the groups immunized with 10^8^, 10^7^, or 10^6^ TCID_50_/mL. However, viral copy numbers were observed on challenge days 3 and 9 in both the 10^5^ TCID_50_/mL low-dose immunized group and the unvaccinated control group, with the unvaccinated control group exhibiting more severe viremia (Fig. 4e). Consistent with the findings on viremia, the duration of viral shedding was shorter in ocular and nasal secretions of the immunized groups compared to the unvaccinated control group. Notably, no detectable viral shedding was observed in the eyes after day six and in the nasal passages after day nine in the immunized groups. Furthermore, the viral load in the eyes on day six and in the nasal on day 9 of the unvaccinat passagesed group was significantly higher than that in the immunized group (Fig. 4f and g). Anatomical examination revealed that the lungs of the non-immunized group exhibited diffuse hyperemic discoloration with multifocal petechial hemorrhages. In contrast, the lungs of the immunized group maintained characteristic pink-tan parenchyma without detectable ecchymosis or congestion (Fig. 5a). The viral load in lung, trachea, nasal turbinate, and throat tissues was assessed. As illustrated in Fig. 5b, the viral load in the tissues of the unvaccinated control group was higher compared to both immunized groups (10^8^ and 10^6^ TCID_50_/mL). Furthermore, viral replication was observed exclusively in the nasal turbinates and lungs of the unvaccinated control group. Histopathological analysis demonstrated that the immunized groups showed no significant tissue damage post-challenge, except for mild submucosal edema in the trachea of the 10^5^ TCID_50_/mL immunization group. Conversely, the unvaccinated control group exhibited substantial inflammatory cell infiltration within the lungs, accompanied by epithelial cell proliferation, resulting in marked thickening of the alveolar septa and perivascular edema. Additionally, there was evidence of tracheal submucosal edema with inflammatory cell infiltration, as well as partial degeneration and necrosis of the mucosal epithelial cells. The nasal turbinates exhibited minor inflammatory cell infiltration and localized edema within the mucosal layer. Furthermore, focal edema of the mucosal propria in the throat was observed, accompanied by inflammatory cell infiltration (Fig. 5c and d). Meanwhile, pathological scores of these tissues were calculated (Fig. 5e), suggesting that the immunization reduces FCV pathological damage to tissues. These findings suggest that rHBDL2 FCV-△VP2 provides robust immune protection in felines and may serve as a promising candidate for a vaccine strain against FCV.

### rHBDL2 FCV-△VP2 and rHBDL2_QD2 VP1_ FCV-△VP2 can produce broadly neutralizing antibodies against different FCV strains

**Fig 6:**
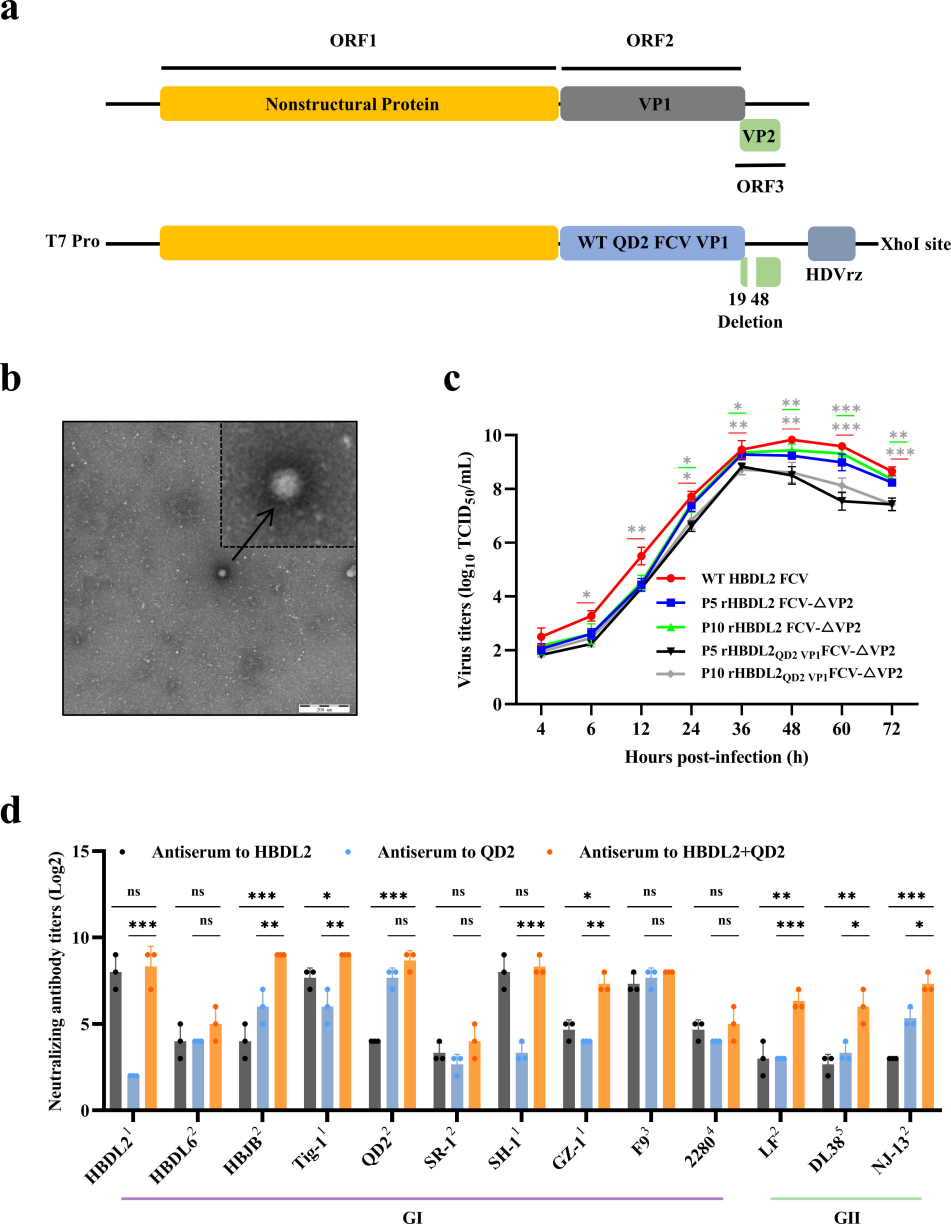
Rescue and characterization of rHBDL2_QD2 VP1_ FCV-△VP2. (**a**) Schematic diagram of the construction of the infectious clone of rHBDL2_QD2 VP1_ FCV-△VP2. (**b**) Transmission electron microscopy was used to observe the morphology of rHBDL2_QD2 VP1_ FCV-△VP2 viral particles, as indicated by black arrows. (**c**) Viral titers (TCID_50_) were assessed at 4, 6, 12, 24, 36, 48, 60, and 72 h post-infection to construct a growth curve for the p5 and p10 generations of rHBDL2_QD2 VP1_ FCV-△VP2 (MOI = 0.001) in F81-VP2 cells. The color-coded asterisk (*) and underline (_) denote comparisons between viral strains. (**d**) The double-vaccinated and single-vaccinated serum neutralizing antibody titers to various FCV isolates (1, VS-FCV strains; 2, respiratory symptoms strains; 3, attenuated vaccine strain; 4, highly virulent strain for inactivated vaccine; 5, non-virulent strain). Statistical analyses were conducted via one-way ANOVA with Tukey’s multiple comparison test (**c**) and unpaired *t*-tests (**d**), with significance levels denoted as follows: *P <  0.05, **P < 0.01, ***P < 0.001.

In our preliminary research, we found that the serum of HBDL2-immunized cats showed a suitable cross-neutralizing response against other FCV strains, especially the VS-FCV strain, but had a weaker neutralizing response against common strains. To increase the protective efficacy of the vaccine candidate against various strains, we replaced the VP1 protein of rHBDL2 FCV-△VP2 with that of QD2 (Fig. 6a). FCV QD2 strain could cause common respiratory symptoms and exhibits low cross-reactivity with FCV HBDL2. According to the transmission electron microscopy results, the rHBDL2_QD2 VP1_ FCV-△VP2 exhibited a morphology comparable to that of classical FCV virions (Fig. 6b). Growth curve analyses of the P5 and P10 generations demonstrated a slightly reduced replication capacity compared with that of both WT HBDL2 FCV and rHBDL2 FCV-△VP2, with the viral titer reaching 10^8.72^ TCID_50_/mL (Fig. 6c). Six cats were subsequently divided into two immunization groups: one received 1 mL of rHBDL2_QD2 VP1_ FCV-△VP2 with 10^6^ TCID_50_/mL and the other was co-immunized with 1 mL (containing 10^6^ TCID_50_/mL) each of rHBDL2 FCV-△VP2 and rHBDL2_QD2 VP1_ FCV-△VP2. The immunization protocol was performed as previously described. After 42 days, serum was collected from blood samples for a virus neutralization assay. Compared with that of single-strain immunization, the cross-neutralizing response of the serum from co-immunized cats to other strains was significantly greater, especially with GII strains with low neutralizing antibodies in serum from cats immunized against a single strain. Notably, the neutralizing antibodies generated by co-immunization exhibit potent cross-neutralizing activity against diverse FCV strains (GI and GII), including VS-FCV strains, respiratory symptom-causing strains, vaccine strain, and non-virulent strain (Fig. 6d). In summary, co-immunization of these two replication-defective viruses can generate broadly neutralizing antibodies, holding promise as a multivalent vaccine strategy for the prevention of FCV infection.

### rHBDL2 FCV-△VP2_mCherry_ has suitable stability and potential as an FCV viral vector vaccine

**Fig 7:**
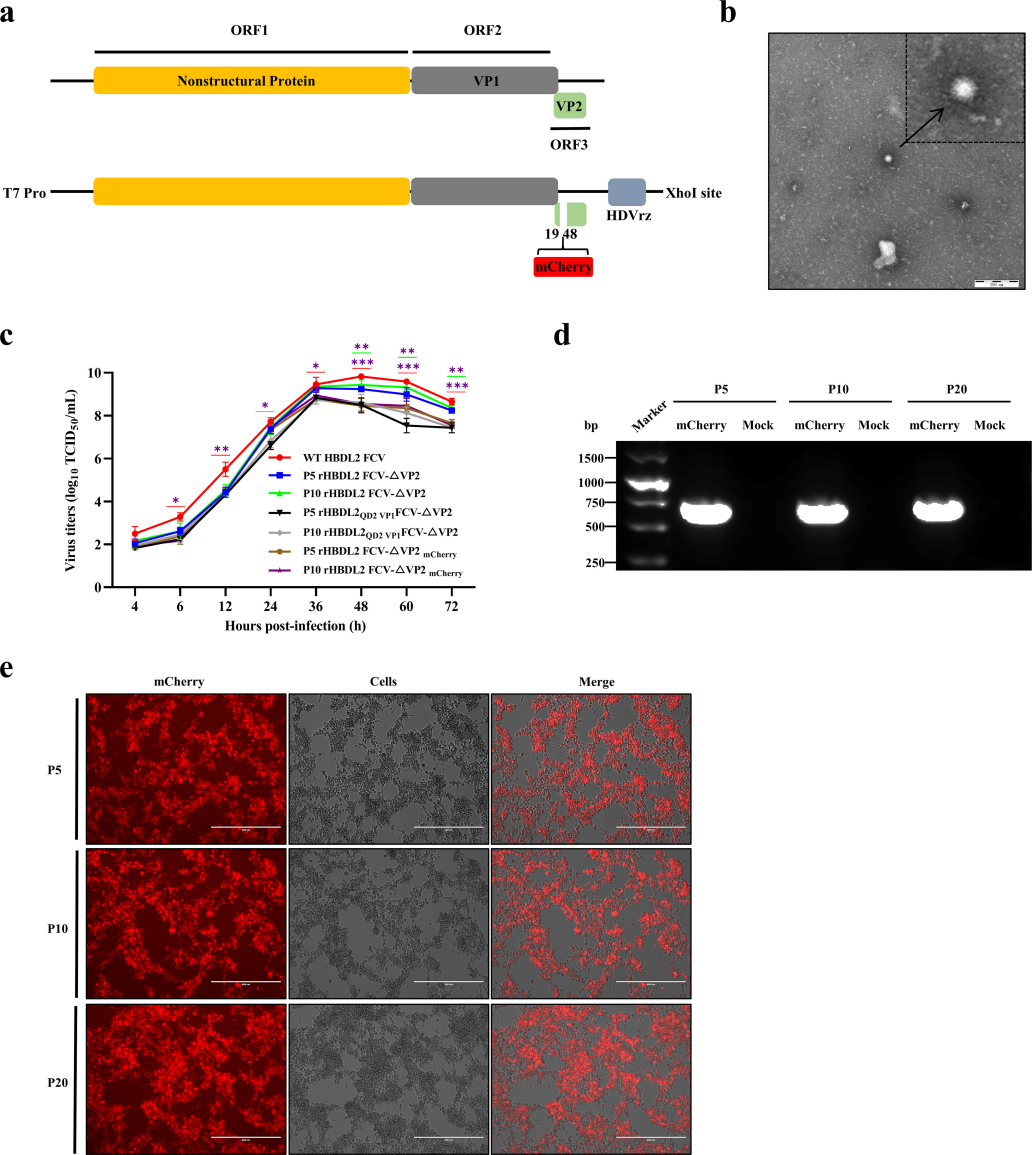
rHBDL2 FCV-△VP2_mCherry_ demonstrated suitable stability and potential as an FCV viral vector vaccine. (**a**) Schematic diagram of the construction of the infectious clone of rHBDL2 FCV-△VP2_mCherry_. (**b**) Transmission electron microscopy was used to observe the morphology of the rHBDL2 FCV-△VP2_mCherry_ viral particles, as indicated by the black arrows. (**c**) p5 and p10 rHBDL2 FCV-△VP2_mCherry_ (MOI = 0.001) were used to infect the F81-VP2 cell line, and the viral titer (TCID_50_) was measured at 4, 6, 12, 24, 36, 48, 60, and 72 h post-infection to plot the growth curve. The color-coded asterisk (*) and underline (_) denote comparisons between viral strains. (**d**) PCR detection of the mCherry gene in the genomes of p5, p10, and p20 rHBDL2 FCV-△VP2_mCherry_ was performed, with the negative control being the rHBDL2 FCV-△VP2 genome. (**e**) The p5, p10, and p20 generations of rHBDL2 FCV-△VP2_mCherry_ (MOI = 0.001) were used to infect the F81-VP2 cell line, and mCherry expression was observed. Statistical analysis was conducted via one-way ANOVA with Tukey’s multiple comparison test, with significance levels denoted as follows: *P  <  0.05, **P  < 0.01, ***P < 0.001.

Moreover, this study investigated the potential for incorporating exogenous genes into replication-defective FCVs as vector vaccines for the development of multivalent and combination vaccines. We incorporated the mCherry red fluorescent gene at the N-terminal deletion site of the rHBDL2 FCV-△VP2 VP2 gene (Fig. 7a) and successfully rescued the virus rHBDL2 FCV-△VP2_mCherry_ through reverse genetics. This modified virus expresses mCherry red fluorescence during the infection of F81-VP2 cells. Negative staining revealed that the morphology of the virions was consistent with the classical FCV structure (Fig. 7b). Moreover, the growth curves of the P5 and P10 generations were analyzed, revealing that rHBDL2 FCV-△VP2_mCherry_ replicated less effectively than did rHBDL2 FCV-△VP2, and its replication capacity was similar to that of rHBDL2_QD2 VP1_ FCV-△VP2, with a virus titer reaching 10^8.94^ TCID_50_/mL (Fig. 7c). F81-VP2 cells infected at an MOI of 0.01 maintained the mCherry gene and its expression up to P20 (Fig. 7d and e). Overall, rHBDL2 FCV-△VP2_mCherry_ is stable and shows promise as a fluorescent reporter virus for developing FCV viral vector vaccines.

## DISCUSSION

FCV as a primary pathogen in domestic and wild felids has long been recognized as a cause of feline upper respiratory tract disease and oral ulceration (28). In recent years, FCV strains have undergone ongoing evolution and mutation, necessitating the timely development and optimization of vaccine candidates. Currently, the approved vaccines for FCV prevention are conventional, comprising live attenuated and inactivated (killed) vaccines (29). However, inactivated vaccines exhibit suboptimal efficacy and are associated with adverse reactions, whereas live attenuated vaccines pose safety concerns due to potential pathogenicity risks (30). Replication-deficient vaccines offer a comparable safety profile to inactivated vaccines and express viral antigens during a single replication cycle within infected cells. This approach combines the safety advantages of inactivated vaccines with the immunogenic benefits of live vaccines, thereby effectively enhancing the host immune response (31). The development of replication-deficient FCV vaccines has consequently garnered our interest. It is prudent to focus on engineering modifications of the FCV VP2 gene, as targeting non-structural genes for such modifications may be inadvisable. This is due to the critical roles played by FCV non-structural proteins in various stages of the viral life cycle, including genome replication and translation. Notably, the FCV non-structural proteins p32, p39, and p30 have been reported to localize to the endoplasmic reticulum, where they initiate the formation of replication complexes (32). Additionally, host proteins such as p53 and A2 have been shown to associate with these viral replication complexes, which are required for efficient replication of FCVs (25, 33). The translation of gRNA and subgenomic RNA is facilitated by the RNA-linked viral protein VPg (34–36). The deletion of non-structural genes is expected to inhibit the expression of gene products within cells. The capsid of the FCV virion is composed of 180 units of the major structural protein VP1 and 12 units of the minor structural protein VP2 (22). Presently, neutralizing epitopes of FCV are located within the hypervariable region of the major capsid protein VP1 (37, 38). The assembly of the FCV virion requires only a limited number of VP2 protein copies, which is advantageous for the development of replication-deficient FCVs. An investigation of the FCV VP2 protein revealed that the VP2 protein facilitates the release of its genome by perforating the endosomal membrane. The hydrophobic amino acid region spanning residues 4–11 at the N-terminus of the VP2 protein is integral to this function (2, 22). Additionally, the 3′ end nucleotide sequence of the VP2 gene contains a *cis*-acting RNA element essential for generating infectious virions. Notably, infectious viral particles were successfully rescued from a full-length FCV cDNA clone carrying a 5′-terminal deletion in the VP2 gene (minimum functional deletion: nucleotides 19–48) through *trans*-complementation with VP2 expressed from a eukaryotic plasmid (26). On the basis of these findings, we successfully engineered rHBDL2 FCV-△VP2, a replication-deficient FCV vaccine candidate characterized by a deletion of 19–48 nucleotides at the N-terminus of the VP2 gene.

Next, we evaluated the potential of the replication-defective virus rHBDL2 FCV-△VP2 as a vaccine candidate. The findings were both anticipated and unexpected. Initially, we successfully produced rHBDL2 FCV-△VP2 at high viral titers, exceeding 10^9^ TCID_50_/mL, in F81 cells that stably expressed the VP2 protein. To confirm that rHBDL2 FCV-△VP2 could infect conventional F81 cells and complete a single cycle of replication, we employed transmission electron microscopy, Western blot analysis, and real-time PCR. Our results demonstrated that the VP2 protein with the N-terminal deletion was effectively involved in the assembly of virions, resulting in the formation of structurally mutilated virions, namely, inc.rHBDL2 FCV-△VP2. Upon reinfection with conventional F81 cells, the viral genome was unable to replicate because of the N-terminal deletion in the VP2 protein, resulting in a replication-defective phenotype. Previous studies have revealed that the VP2 protein can penetrate the endosomal membrane to facilitate genome release. This finding further elucidates the role of FCV VP2 in the replication of infectious virions.

In subsequent experiments, our data demonstrated that rHBDL2 FCV-△VP2 could provide strong immune protection in cats. Immunized cats produced high levels of neutralizing antibodies, exhibited no organ invasion, demonstrated a favorable safety profile, and showed a significant reduction in clinical symptoms following challenge exposure. Notably, no viremia was detected in cats immunized with 10^8^, 10^7^, or 10^6^ TCID_50_/mL, and a shorter duration of viral shedding and a lower organ viral load were observed in the immunized groups. Additionally, histopathological analysis of the respiratory-related tissues revealed no damage across all immunized groups, which significantly differed from that in the unvaccinated control group. These findings indicate that rHBDL2 FCV-△VP2 holds promise as an effective and safe novel FCV vaccine candidate.

Furthermore, to broaden the neutralizing effects of the serum from vaccinated cats and enhance the protective efficacy of the vaccine candidate against various strains, we replaced the VP1 region of the vaccine candidate FCV HBDL2 with that of the QD2 strain, which induces common respiratory symptoms and exhibits limited cross-reactivity with HBDL2. Compared with immunization with rHBDL2 FCV-△VP2 alone, immunization with both VP1 candidates resulted in the production of broadly neutralizing antibodies. Interestingly, specific strains exhibit limited cross-reactivity when immunized individually with either of the two strains. However, they demonstrate high cross-reactivity upon co-immunization. These findings indicate that a vaccine candidate containing rHBDL2 FCV-△VP2 and rHBDL2_QD2 VP1_ FCV-△VP2, which exhibit significant VP1 diversity, can induce a broad spectrum of antibodies against various strains, thereby increasing the efficacy of the FCV vaccine.

Finally, we investigated the potential application of replication-defective recombinant viruses as viral vectors for the expression of exogenous genes in the development of multivalent vaccines. We attempted to incorporate the mCherry gene into the deleted VP2 region of the replication-deficient FCV. Fortunately, a genetically stable red fluorescent recombinant virus that can continuously emit red fluorescence in infected cells was successfully generated. These findings validate the effective incorporation of foreign genes into the deleted FCV VP2 region, underscoring its potential for the development of viral vector vaccines. A recent study supported our findings by demonstrating the successful propagation of a recombinant MNV containing exogenous reporter genes within the non-essential region of ORF3 facilitated by the provision of VP2(27). Moreover, FCV serves as an exemplary model for investigating the biology of caliciviruses and can be utilized as a reporter virus. These findings may facilitate the exploration of the molecular mechanisms underlying the effects of FCV and other caliciviruses.

Grounded in the study of human‒animal interactions, the health of companion animals holds substantial significance for human physical and mental well-being (39). FCV, a primary pathogen, poses a serious threat to the health of feline populations. In this study, we successfully developed a replication-deficient FCV, designated rHBDL2 FCV-△VP2, and assessed its potential as a novel vaccine. The results demonstrated that immunization with this vaccine conferred robust immune protection against VS-FCV challenge in cats and significantly mitigated clinical symptoms. Furthermore, the adaptability of this replication-defective FCV platform holds potential for the development of viral vector vaccines as well as multivalent vaccines.

## MATERIALS AND METHODS

### Plasmid, cells, and viruses

The pLVX-VP2 His-IRES-ZsGreen1 recombinant plasmid, optimized for commercial codon synthesis (GenScript, Nanjing, China), was utilized in this study. Additionally, the pMD2.G, psPAX2, and pOK12 plasmids were maintained in our laboratory. Feline kidney cells (F81) and human embryonic kidney cells (293T), along with F81-VP2 cells (F81 cell line with stable expression of the VP2 gene), were developed via a lentiviral packaging system. These cell lines were cultured in Dulbecco’s modified Eagle’s medium (DMEM; Gibco, Waltham, MA, USA) supplemented with 10% fetal bovine serum (FBS; Clark Bioscience, Richmond, VA, USA) and incubated at 37°C in a 5% CO_2_ atmosphere. The FCV HBDL2 strain was isolated in 2021 from clinical samples obtained from a cat exhibiting severe systemic symptoms in Heilongjiang Province. The FCV QD2 strain originated from 2021 clinical samples collected from a cat with respiratory manifestations in Shandong Province. Reference strains FCV 2280 and F9 were acquired from the American Type Culture Collection. Additional FCV strains included in this study had been previously isolated, characterized, and stored in our laboratory (40, 41). All viral stocks were propagated in either F81 cells or genetically modified F81-VP2 cells.

### Cell line generation

The cell lines were developed via a lentiviral packaging system comprising pLVX-VP2 His-IRES-ZsGreen1, pMD2.G, and psPAX2. These components were transfected into monolayer 293T cells using the Lipofectamine 2000 Transfection Reagent (Thermo Fisher Scientific, Massachusetts, MA, USA). Following a 48 h incubation period, the supernatant containing the viral particles was collected and used to infect a monolayer of F81 cells. The cells were subsequently sorted on the basis of green fluorescence, resulting in the establishment of the F81-VP2 cell line, which was achieved by obtaining a positive rate exceeding 95% on two separate occasions.

### Construction of recombinant cDNA clones

The WT HBDL2 FCV genome was segmented into four fragments: A (2,459 bp), B (2,892 bp), C (2,046 bp), and D (450 bp). A targeted deletion was performed on nucleotides 19–48 of the VP2 gene. The T7 promoter was introduced at the 5′ end of fragment A via PCR, while the polyadenylation signal and HdvRz were appended to the 3′ end of fragment D. Linearized pOK12 was generated through PCR. Each of the five fragments, A–D, along with the linearized pOK12, contained 20 bp homology arms. Recombinant rHBDL2 FCV-△VP2 cDNA clones were subsequently constructed via homologous recombination. Using a similar strategy, the rHBDL2 FCV-△VP2 VP1 gene was substituted with the WT QD2 FCV VP1 gene, resulting in the generation of the rHBDL2_QD2 VP1_ FCV-△VP2 cDNA. Additionally, the mCherry red fluorescent gene was inserted at the site corresponding to the deletion of nucleotides 19–48 from the N-terminus of rHBDL2 FCV-△VP2, thereby producing the rHBDL2 FCV-△VP2_mCherry_ cDNA. In this investigation, a site-directed mutation was introduced at nucleotide position 136 of the FCV cDNA clones, altering it from C to G to serve as a genetic marker distinguishing the recombinant viruses from the WT strains.

### Rescue of recombinant FCV

Recombinant FCV cDNA clones were linearized through digestion with XhoI (Thermo Fisher Scientific). The linearized cDNA was subsequently transcribed *in vitro* into capped RNA via the HiScribe T7 High Yield RNA Synthesis Kit and Cap Analogs (New England Biolabs, Beijing, China). The residual cDNA was eliminated via the use of RNase-free DNase I (New England Biolabs). The capped RNA was purified via the RNeasy Mini Kit (Qiagen, Valencia, CA, USA) and subsequently transfected into a monolayer of F81-VP2 cells using Lipofectamine 2000. The recombinant FCV virus was harvested 48 h post-transfection.

### Virus titration and *in vitro* growth curve

To determine the TCID_50_, F81-VP2 cells were cultured as monolayers in 96-well plates for 24 h. FCV, which was serially diluted 10-fold 11 times in DMEM, ranging from 10^1^ to 10^11^, was then inoculated into F81-VP2 cells. Following a 1 h virus adsorption period at 37°C, the virus dilutions were removed, and the cells were washed three times with DMEM. The medium was then replaced with fresh DMEM supplemented with 1% FBS. CPE was monitored after incubation at 37°C for 48 h. The TCID_50_ was subsequently calculated via the Reed‒Muench method.

F81-VP2 cells were cultured as monolayers in 12-well plates for 24 h to assess the growth curve. The cells were subsequently infected with FCV at an MOI of 0.001 and incubated at 37°C. Post-infection, the cells were washed three times with DMEM supplemented with fresh DMEM containing 1% FBS. The cell lysates and supernatants were collected at 4, 6, 12, 24, 36, 48, 60, and 72 h post-infection to determine the TCID_50_.

### Transmission electron microscopy

The virus suspension was added dropwise onto copper grids. The grids were subsequently blotted with filter paper and subjected to negative staining with a 2% (wt/vol) phosphotungstic acid solution for 45  s, followed by a second blotting with filter paper to ensure dryness. Images were then acquired via transmission electron microscopy.

### Western blotting

The cells were cultured as monolayers in 12-well plates for 24 h. Subsequently, the cells were infected with FCV at 37°C. Following a 1 h adsorption period, the supernatant was removed, and the cells were washed three times with DMEM. The medium was then supplemented with fresh DMEM containing 1% FBS. Post-infection, either the supernatant or the cells were collected. The cells were lysed at low temperatures in radioimmunoprecipitation assay (RIPA) buffer (Beyotime Biotechnology, Shanghai, China), and the resulting lysates were centrifuged at 12,000 rpm for 10 min to eliminate precipitates. Equal volumes of the lysates were subjected to 12% SDS-PAGE and subsequently transferred onto polyvinylidene difluoride membranes with a pore size of 0.2 µm (Millipore, Darmstadt, Germany). After being blocked with 5% skim milk, the membranes were incubated overnight at 4°C with laboratory-preserved murine anti-VP1 monoclonal antibody (dilution 1:2,000) and murine anti-β-actin monoclonal antibody (dilution 1:2,000; Abbkine, Wuhan, China). The membranes were then incubated with Dylight 800 goat antimouse IgG (dilution 1:15,000; Abbkine) at room temperature for 1 h. The results were visualized using an Odyssey CLx infrared fluorescence scanning imaging system. Quantitative analysis was conducted by evaluating the grayscale values of the VP1 protein band and the internal reference β-actin protein band via ImageJ software, as needed.

### Quantitative real-time PCR

Total RNA was isolated using an RNeasy Mini Kit (Qiagen). Gene-specific primers for FCV (a 135 bp conserved region spanning the P76-VP1 junction) and β-actin were designed via SnapGene software. The primer sequences for FCV were as follows: forward, 5′-AGTTTAATGGTGTGGAGACGCG-3′; reverse, 5′-TGGGGATCCCAATCATAGTA TTT-3′. For β-actin, the sequences of primers used were as follows: forward, 5′-CAGGTGATCACCATCAGGCAAC G-3′; reverse, 5′-GACAGCACCGTGTTAGCGTAGAGGT-3′. Relative quantification was conducted using a UniPeak U + One Step RT‒qPCR SYBR Green Kit (Vazyme Biotech, Nanjing, China). β-Actin mRNA served as an internal reference for normalization, and the relative mRNA levels of FCV were determined via the 2^−△△CT^ method. Probes targeting FCV-specific primers were synthesized commercially as needed. Absolute quantification was conducted via the AccurSTART U + One Step RT‒qPCR Probe Kit (Vazyme Biotech). The FCV copy number was determined using a standard curve.

### Vaccination and virus challenge

The experimental cats were supplied by the National Engineering Research Center of Veterinary Biologics Corp. (Harbin, China). Briefly, the cats were negative for FCV, parvovirus, herpesvirus, and infectious peritonitis virus, as determined by RT-PCR or PCR. Twenty-six domestic cats, aged 2 months and weighing between 0.75 and 1.0 kg, were randomly assigned to five groups. Four groups were immunized with rHBDL2 FCV-△VP2 (10^8^, 10^7^, 10^6^, and 10^5^ TCID_50_/mL). An additional cat was included in the 10^8^ TCID_50_/mL group for safety assessment purposes. The remaining group served as the unvaccinated control group. The immunized groups were immunized twice with an interval of 21 days between each immunization. Three weeks after the boost immunization, one cat in the 10^8^ TCID_50_/mL group was randomly selected for euthanasia via intravenous administration of 20% sodium pentobarbital (0.3 mL/kg), in accordance with the protocol recommended by the World Society for the Protection of Animals, as detailed in the “Methods for the Euthanasia of Dogs and Cats” guide. RNA was extracted from the trachea, heart, liver, spleen, lung, and kidney through tissue homogenization. FCV VP1 gene-specific primers were designed for PCR detection via SnapGene software. The FCV sequences were as follows: forward: 5′- ATGTGCTCAACCTGCGCTAACGTGCTAAAA-3′; reverse: 5′-CATAATTTAGTCATAACACTCCTAATATTT-3′.

Challenge experiments were performed immediately after the end of immunization. The cats were inoculated with 0.5 mL (0.2 mL for each nasal passage and 0.05 mL for each eye) of 10^8^ TCID_50_/0.5 mL WT HBDL2 FCV via the intranasal and ocular routes. The clinical symptoms were recorded daily, and the clinical scores, which included respiratory, oral cavity, and eye scores, were assessed on a scale of 0–3 (Table 1) (1). The clinical scoring process was performed in a double-blind (participant and assessor) manner to avoid significant bias introduced to the clinical scoring. The challenge cycle was 15 days. In addition to daily observations of clinical symptoms, body temperature and body weight were measured on days 0, 3, 6, 9, 12, and 15 post-inoculation. Anticoagulation samples were collected on days 0, 3, 9, and 15 post-inoculation. Blood viral load (copy number) was measured via RT-qPCR to determine viremia. Additionally, the swabs in the eye and nose of 10^8^ and 10^6^ TCID_50_/mL and the unvaccinated control groups were collected for detecting the viral shedding. On day 15, three cats from each challenge group were euthanized by intravenous injection of 20% sodium pentobarbital (0.3 mL/kg) according to the protocol recommended by the World Society for the Protection of Animals, Methods for the Euthanasia of Dogs and Cats. Lung, trachea, nasal turbinates, and throat samples were harvested for histological analysis and viral load testing. During the experiment, the challenged cats were humanely euthanized when they were observed to be in pain, were not moving, and lost the ability to eat and drink.

**TABLE 1:** Standard for assessing clinical signs

Score	Depression and anorexia	Oral cavity symptoms	Respiratory symptoms	Ocular discharges	Lameness
0	No symptoms	no symptoms	No symptoms	No symptoms	No symptoms
1	Depression	One little ulcer spot (diameter <0.5 cm)	Sneezing (one to two times per 10 min)	Clear secretion (one eye)	Walking posture deformation and ability to bear weight on the affected foot
2	1/3–1/2 food intake	Two to three little ulcer spots (diameter <0.5 cm)	Sneezing (one to three times per 5 min)	Clear secretion (two eyes)	Reluctance to bear weight on the affected foot and unwillingness to place weight on the affected limb, sitting with the limb off the ground
3	Apatia	Big ulcer spots (diameter >1 cm)	Mouth breathing and wheezing	Purulent secretion	Disability to bear weight on the affected foot and trouble walking and rising

### Virus neutralization assays

For the virus neutralization assay, serum was isolated and inactivated at 56°C for 30 min. The serum was then subjected to a twofold serial dilution using DMEM, ranging from 1:2^1^ to 1:2^12^. Subsequently, 60 µL of the diluted serum was combined with 60 µL of the virus at a concentration of 200 TCID_50_ and incubated at 37°C for 1 h. The virus–serum mixtures were then added to F81 cells that had been washed with PBS and incubated at 37°C with 5% CO_2_ for 48 h. Four replicates were prepared for each serum dilution. No cytopathic effects were observed, indicating the ability of the serum to neutralize the virus.

### Histopathology

Lung, trachea, turbinates, and throat samples were obtained on the 15th day following WT HBDL2 FCV challenge and subsequently fixed in 4% paraformaldehyde for 2 days. The tissues were subjected to gradient alcohol dehydration and clearing before being embedded in paraffin. They were then sectioned into 5 µm-thick slices. These sections were stained with hematoxylin and eosin (HE) to facilitate histological examination.

### Statistics

The data are presented as the mean ± standard deviation (SD). Statistical significance was assessed via unpaired *t*-tests or one-way analysis of variance (ANOVA) with Tukey’s multiple comparison test conducted with Prism version 9.3.0 software (GraphPad Software), with a value of *P* < 0.05 considered indicative of a significant difference.

## Data Availability

All data needed to evaluate the conclusions in the paper are present in the paper, and additional data related to this paper are available upon request.
